# Preparation and characterization of novel MWCNTs/Fe-Co doped TNTs nanocomposite for potentiometric determination of sulpiride in real water samples

**DOI:** 10.1038/s41598-020-65592-y

**Published:** 2020-05-25

**Authors:** M. M. Khalil, A. A. Farghali, Waleed M. A. El Rouby, I. H. Abd-Elgawad

**Affiliations:** 1 Chemistry Department, Faculty of Science, Beni-Suef University, Beni-Suef, Egypt; 2 Materials Science and Nanotechnology Department, Faculty of Postgraduate Studies for Advanced Science, Beni-Suef University, Beni-Suef, Egypt

**Keywords:** Biochemistry, Sensors

## Abstract

Novel multiwalled carbon nanotubes/ Fe-Co doped titanate nanotubes nanocomposite (MWCNTs/Fe-Co doped TNTs) facilitated the charge transfer and enhanced sensitivity and selectivity. Herein, three novel modified carbon paste sensors (CPSs) based on MWCNTs (sensor I), Fe-Co doped TNTs (sensor II) and MWCNTs/Fe-Co doped TNTs composite (sensor III) were fabricated for a simple, low cost and high accuracy electrochemical method for the potentiometric determination of sulpiride (SLP). The sensors exhibited excellent Nernstian slopes 57.1 ± 0.4, 56 ± 0.5 and 58.8 ± 0.2 mV decade^−1^ with detection limits (DL) 7.6 × 10^−7^, 1.58 × 10^−6^ and 8.7 × 10^−8^ mol L^−1^, quantification limits (QL) 2.5 × 10^−6^, 5.2 × 10^−6^ and 2.9 × 10^−7^ mol L^−1^ for a long lifetime 20, 18, and 25 weeks for sensors (I), (II), and (III), respectively. The modified sensor (III) was applicable by measuring the concentration of spiked SLP in pure solutions, pharmaceutical products, human urine, and real water samples. The proposed method can be used as an important analytical tool in the quality control of the pharmaceutical industry.

## Introduction

According to IUPAC recommendation, SLP named 5-(aminosulfonyl)-N-[(1-ethylpyrrolidin-2-yl)methyl]-2methoxybenzamide (C_15_H_23_N_3_O_4_S) is the most widely prescribed anti-psychotic drug^1^. SLP may be more effective than other older drugs for treatment of acute and chronic schizophrenia^2^.

Various methods have been used for SLP determination including spectrophotometry^3^, electrophoresis^4,5^, adsorptive stirring voltammetry^6^, fluorimetry^7^, flow injection chemiluminometry^8^, thin layer chromatography-densitometry^9^, high performance-liquid chromatography^3,9,10^ and liquid chromatography-mass spectrometry^11^. The majority of these methods include one or more defects such as narrow linear range (LR)^5,7,9^, low sensitivity and robustness for biological samples^4^ and time consuming^3,10,11^. Therefore, our goal aimed to avoid wasting time, cost and sensitivity for SLP micro determination.

Electrochemical methods have good accuracy, precision, and low cost. For instance, potentiometry plays an important role in sensing and determination of drugs in samples. It has numerous advantages such as easy fabrication, high selectivity and rapid determination^12^. CPSs were first published by Mesaric and Dahmen^13^. These sensors have unique advantages including: renewability, simplicity in the assembly, chemical inertness, stability, high resistance, environmentally friendly and without internal filling solution^14^. Furthermore, CPS can be easily modified with new sensing and conducting materials to enhance the sensor potentiometric response.

Cyclodextrins (CDs) are used for modifying the sensing electrodes to improve sensitivity and selectivity^15,16^. This can be attributed to the formation of what is called “inclusion complexes”^17^.

Modification of potentiometric sensors in order to enhance sensitivity, selectivity and lowering DL was achieved using nanomaterials due to their distinguished properties^17^. This material has a highly porous hollow structure, excellent low resistance, and large specific surface area^18^. Recently, Darzi and Shajie^19^ reported that nano - TiO_2_ was applied successfully for technological applications. However, the electrical conductivity of TNTs is very low which can affect negatively on the sensor response. Thus, increasing the conductivity of the sensor will improve the response time and the operating concentration range^20^. For that reason, the conductivity of TNTs can be enhanced by doping with transition metals^21–23^.

Tong *et al*.^24^ demonstrated high sensitivity, shorter response and recovery time of Co doped TNTs based sensor for H_2_S gas. The transition metal Fe doped TNTs enhanced stability, absorption and photoelectrochemical activities as compared to TNTs alone^25^. In this work, Fe and Co were utilized as dopants for TNTs to enhance conductivity, electron transfer, surface area, thermal stability and photoelectrocatalysts; to reduce the energy gap or energy levels by incorporating Fe and Co ions into crystal lattices^26,27^.

Another solution for the previously mentioned problem is the combination of TNT/CNTs in one nanocomposite where the stability and surface area in addition to the electrical conductivity can be enhanced^28,29^. MWCNTs and TiO_2_ nanocomposite was applied as better signal transducers to improve the performance of CPS for hyoscine butylbromide determination^30^. Also, the above mentioned nanocomposite was incorporated in modified CPS achieving high sensitivity, stability and long lifetime for Thallium(I) micro determination as reported by Bagheri *et al*.^31^. Abdallah and Ahmed^32^ constructed new and sensitive CPS based on MWCNTs/ TiO_2_ nanocomposite for potentiometric determination pazufloxacin. Recently, MWCNTs and Ru doped TiO_2_ were well utilized for clozapine determination which improved the potentiometric sensor sensitivity and minimized the DL^33^ compared with Ru doped TiO_2_ alone^34^.

For maximizing the utilization of CNTs and transition metals doped TNTs properties, our vision was devoted to use a nanocomposite of both materials. Hence, in this work a new, simple and sensitive electrochemical methodology was developed for SLP determination. Modified CPS with MWCNTs (sensor I), Fe-Co doped TNTs (sensor II) and MWCNTs/Fe-Co doped TNTs nanocomposite (sensor III) were fabricated to achieve a better sensor response. Sensor (III) was successfully used for SLP determination in pharmaceutical formulations, biological and real samples.

## Experimental

### Reagents and materials

All chemicals and reagents used were extra pure. Deionized water was used for all preparation of solutions. Pure-grade SLP (M.wt = 341.4 g mol^−1^) was provided by Sanofi for pharmaceutical industry, Egypt. The pharmaceutical preparation Dogmatil® Fort (SLP, 200 mg per tablet) was purchased from local drug stores. Dioctyl adipate (DOA) and sodium tetraphenylborate (NaTPB) were obtained from Fluka (U.S.A.). Spectroscopic graphite powder (1–2 µm), dibutyl phthalate (DBP), dioctyl sebacate (DOS), acetophenone (AP) and dioctyl phthalate (DOP) were purchased from Merck (Germany). β-cyclodextrin (β-CD), dibenzo-18-crown-6 and 18-crown-6 were purchased from Euromedex (France). The metal salts were provided by BDH as nitrates or chlorides.

A standard solution of 10^−2^ mol L^−1^ SLP was prepared by dissolving an appropriate amount of pure SLP in 0.05 mL of concentrated HCl to form SLPCl and then diluted to 50 mL by deionized water. Other dilute solutions (1.0 × 10^−9^–1.0 × 10^−2^) were prepared by serial dilution and kept at 4 °C.

### Apparatus

702 titroprocessor (Metrohm, Switzerland) was used for potentiometric and pH-measurements. FESEM images were taken by (FEI- Quanta feg-250 SEM, Switzerland) for the sensor paste. HRTEM images were recorded by (Jeol 2100 HRTEM, Japan). Electrochemical impedance spectroscopy (EIS) studies were performed using potentiostat (Autolab PGSTAT 302 N, Netherlands).

### MWCNTs Synthesis

The chemical vapor deposition (CVD) technique was applied for synthesizing MWCNTs with high purity as described in the previously published work^35^ using Co-Fe/CaCO_3_ catalyst/support at 600 °C. The prepared CNTs were first purified from the residual CaCO_3_ and the catalyst particles using diluted HCl followed by treatment with concentrated mixture of H_2_SO_4_/HNO_3_ (3:1 v/v) under reflux condition at 120 °C for 4 h. Then, the treated CNTs were separated, washed several times with deionized water and allowed to dry at 80 °C overnight. Fig. S1 showed the HRTEM of prepared MWCNTs.

### Fe-Co doped TNTs Synthesis

Recently, TNTs were prepared applying the hydrothermal method^36^ in an alkaline medium. An aqueous solution of 5 g TiO_2_ powder in 250 mL of 10 N NaOH was prepared by constantly stirring for an hour. Then, the suspension was transferred to Teflon-lined stainless-steel autoclave followed by heat treated at 160 °C for 23 h to produce sodium titanate nanotubes. The suspension cooled at room temperature, the white precipitate was washed with distilled water and finally dried at 80 °C for 24 h. Second, Fe-Co doped TNTs was synthesized through ions exchange process^37,38^. Briefly, 1 g of Na-titanate powder was added to 150 ml of a mixture of cobalt and ferrous sulfate solution with a proper concentration (3:7 wt ratio). Then, the mixture was sonicated (20 kHz) for 30 min, the samples were filtered, washed with distilled water to adjust the pH, and then dried at 80 °C for 2 h. Finally, Fe-Co doped TNTs were characterized using X-ray diffractometer (XRD), HRTEM and X-ray photoelectron spectroscopy analysis (XPS)^39^.

### Sensors construction

**Figure 1:**
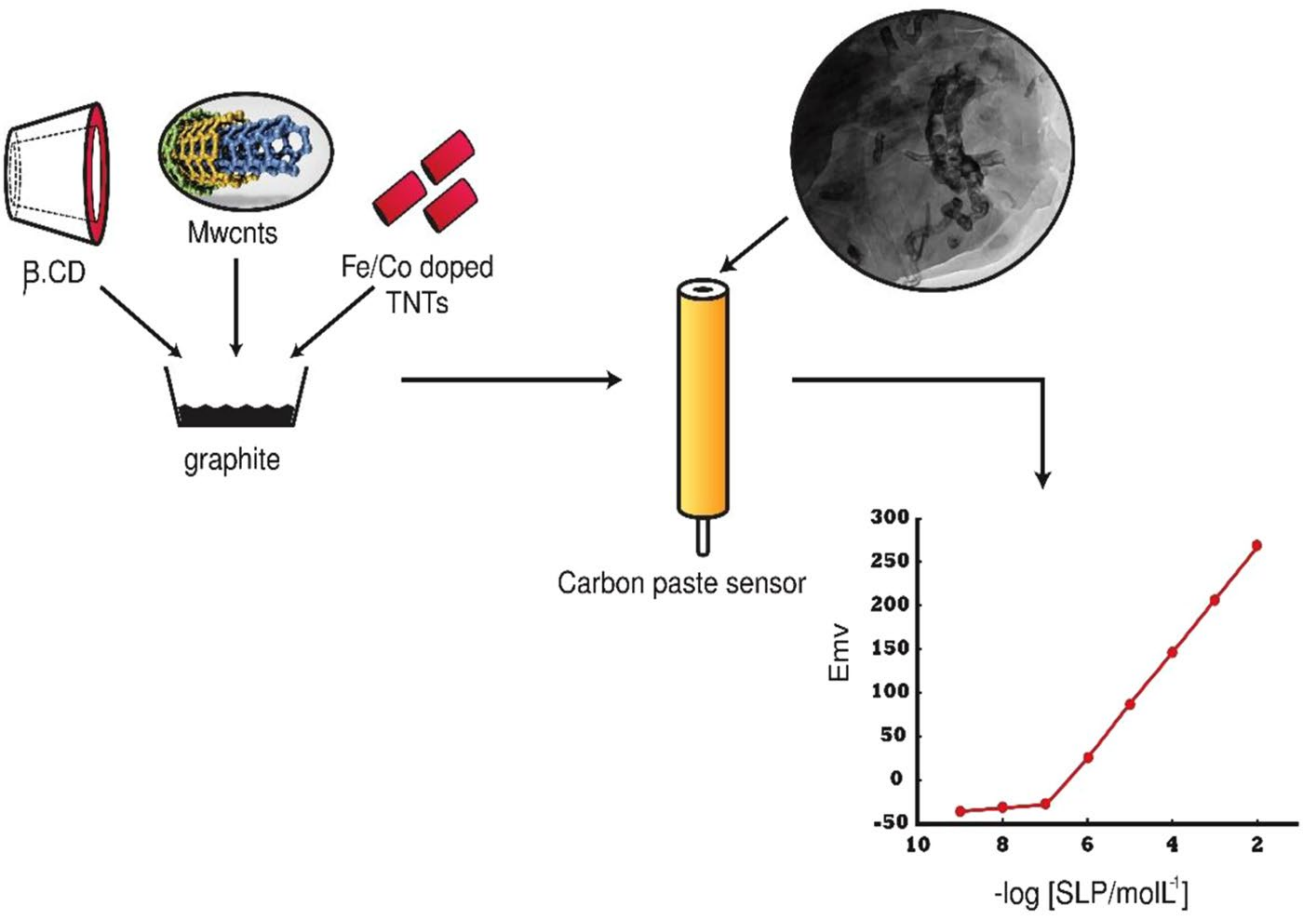
Schematic representation of sensor III development.

Three CPEs were fabricated by mixing β-CD ionophore, (MWCNTs, Fe-Co doped TNTs and MWCNTs/Fe-Co doped TNTs nanocomposite), NaTPB lipophilic anionic additive and DBP as a plasticizer. The strategy for potentiometric sensor construction is shown in Fig. 1.

### SLP micro determination

NaTPB was used as titrant to titrate against different volumes (2-6 mL) of 1.0 × 10^−2^ mol L^−1^ SLP. Standard addition method^40^ was used for micro determination of various concentrations from pure drug, pharmaceutical preparation, real urine and surface water samples.

### Statement

All experiments and methods were performed in accordance with relevant guidelines and regulations. All experimental protocols were approved by a named institutional/licensing committee. Specifically, urine collections and experiments (and relevant protocols) were approved by the Regional Ethics Committee (REC) (2011/1337/REK S-OE D) (Oslo, Norway). Informed consent was obtained from all subjects, and all methods were carried out in accordance with the relevant guidelines and regulations of REC.

## Results and discussion

### Characterization of Fe-Co doped TNTs

According to co-author study^39^, the tubular structure of Fe-Co doped TNTs has been confirmed using HRTEM images. In the previous study, co-author studied different dopant ratio of Fe and Co. The doping process has been confirmed using different tools such as XPS, XRD and diffused reflectance spectroscopy. The ion exchange process between Na and Fe, Co has been confirmed by XPS.

The band gap shift after doping of TNTs with different Fe-Co ratios has been studied.

The band gap for TNT was 3.4 eV while for Fe-Co doped TNTs was 2.1 ± 0.1 eV confirming the band shift after Fe and Co doping. In the current study we have select only one concentration of Fe-Co doped TNT where the Fe:Co ratio was 3:7.

### Optimal sensors matrices compositions

In fact, the application of ion-pair based potentiometric sensors is usually restricted by limited selectivity in complex biological samples. Consequently, the sensitivity and selectivity of potentiometric sensors are improved using ionophores as sensing materials through the formation of inclusion complexes with the target analytes^41^. Our preliminary study based on β-CD ionophore, NaTPB as lipophilic anionic additive and DBP as plasticizer showed slope 59.5 ± 0.3 mV decade^−1^ within the concentration range 1.0 × 10^−5^ – 1 × 10^−2^ mol L^−1^ with DL 5.0 × 10^−6^ mol L^−1^^17^, to enhance the performance for this sensor we modified the sensor paste with novel nanocomposite.

### Ionophores effect

The kinetics and thermodynamics of potentiometric sensors can be improved by using a suitable ionophore. Therefore, different ionophores (β-CD, 18-crown-6, dibenzo 18-crown-6 and natural polymer chitosan) were investigated. The data revealed that the sensor incorporated with β-CD enhanced the sensitivity and selectivity compared with the other tested ionophores as mentioned previously^4,5^. Fig. S2 illustrated that the SLP drug has an aromatic ring system which can have hydrophobic interactions with the CD cavity to form SLP-CD stable inclusion complex. Furthermore, has N-alkyl group can also participate in the hydrogen bonding with CD without changing their structure.

### Solvent mediator effect

The dielectric constant and relatively high mobility of the paste constituents have a great effect on the type of plasticizers used. Various equilibria between β-CD ionophore and SLP in the paste phase can be controlled by using a suitable plasticizer. Therefore, sensor performance can be enhanced^42,43^. Beside the dielectric constant, lipophilicity, viscosity, volatility, molecular weight and cost are considered other important factors affecting the potentiometric response of sensors. Different solvent mediators (DBP, DOS, DOA, DOP, and AP) covering a wide range of dielectric constants (ε_*r*_ 4.0–17.4) were evaluated. The best potentiometric response was achieved for the sensor plasticized with DBP. Consequently, DBP (ε_*r*_ 6.4) is used as a suitable plasticizer for construction of the proposed sensor. It is obvious to note that the bad potentiometric response for the sensor plasticized with AP (ε_*r*_ 17.4) can be ascribed to high volatility and water solubility of this plasticizer.

### Performance enhancement with nanomaterial

Nowadays, nanomaterials play important role in electrochemical sensors in decreasing the resistance and increasing the area of the surface. Therefore, the performance of the sensor can be enhanced. Herein, various nanomaterials including MWCNTs, Fe-Co doped TNTs, and MWCNTs/Fe-Co doped TNTs incorporated with sensing material were tested (Table 1).

**Table 1:** Response characterization of the fabricated sensors.

Parameters	sensor I	sensor II	sensor III
Matrix composition	38.95%Graphite + 49.65%DBP + 0.7% β-CD + 0.7% NaTPB+ 10% MWCNTs	43.95%Graphite + 49.65%DBP + 0.7% β-CD + 0.7% NaTPB+ 5%Fe-Co doped TNTs	33.95%Graphite + 49.65%DBP + 0.7% β-CD + 0.7% NaTPB+10%MWCNTs+5%Fe-Co doped TNTs
Slope (mV decade ^−1^)	57.1	56	58.8
Correlation coefficient (r^2^)	0.999	0.999	0.999
SD of slope (mV decade^−1^)	0.4	0.5	0.2
RSD (%)	0.8	1	0.5
Response time (sec.)	5	7	4
Working pH range	—	—	2-8
LR (mol L^−1^)	1.0 × 10^−6^- 1.0 × 10^−2^	4.0 × 10^−6^–1.0 × 10^−2^	1.0 × 10^−7^–1.0 × 10^−2^
DL (mol L^−1^)	7.6 × 10^−7^	1.58 × 10^−6^	8.7 × 10^−8^
QL (mol L^−1^)	2.5 × 10^−6^	5.2 × 10^−6^	2.9 × 10^−7^
Lifetime (weeks)	20	18	25
Thermal temperature coefficient (V/°C)	—-	—	0.0026

The range of MWCNTs extended from 5 to 15% (w/w relative to carbon powder) was incorporated in the paste containing 0.7% β-CD and 0.7% NaTPB (sensor I). The results indicated that the addition of 10% MWCNTs improved the linear response in the concentration range 10^−6^-10^−2^ with a Nernstian slope 57.1 mV decade^−1^ (Fig. S3a). The presence of MWCNTs in the composition of the sensor (I) increased the transduction of the ion to electron signal, improved conductivity of sensor and also the surface area of the paste^44^. Therefore, the sensitivity and concentration range of sensor were enhanced.

Different ratios of Fe-Co doped TNTs were mixed to 0.7% β-CD and 0.7% NaTPB (sensor II). The results showed that 5% Fe-Co doped TNTs increased the LR to 4.0 × 10^−6^–10^−2^ with DL 1.58 × 10^−6^ and slope 56 mV decade^−1^ (Fig. S3b). This can be attributed to the presence of Fe and Co which improved the conductivity of TNTs. Fe and Co can promote electron transfer between SLP and the sensor surface, which can improve the selectivity and sensitivity for the sensor surface.

**Figure 2:**
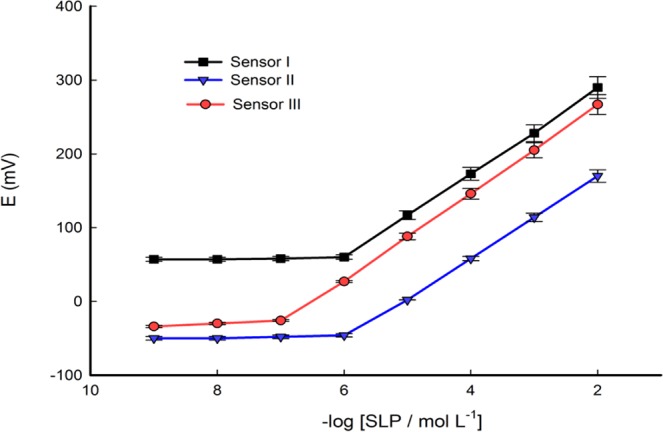
Calibration graphs for sensor І, sensor ІІ and sensor ІІІ at optimum membrane composition.

The addition of 10% MWCNTs and 5% Fe-Co doped TNTs to 0.7% β-CD and 0.7% NaTPB to fabricate the sensor (III) caused an improvement in its concentration LR and DL 10^−7^–10^−2^, 8.7 × 10^−8^ mol L^−1^, respectively (Fig. 2). This may be due to addition MWCNTs to TNTs which increased the surface area and conductivity^45^. Therefore, superior capability for sensors based on nanocomposites is expected. This behavior can enhance the stability, reproducibility and electrocatalytic properties of sensor^46^.

### Characterization of sensors surface morphology

**Figure 3:**
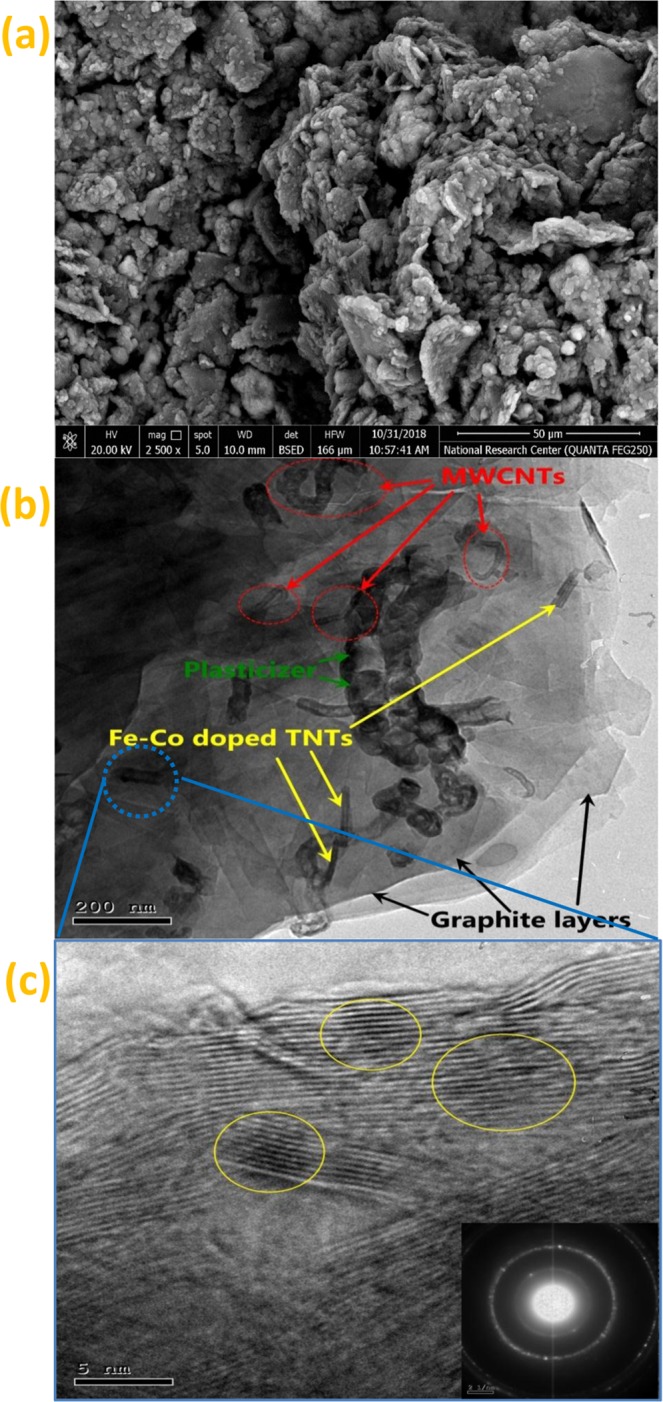
SEM image of MWCNTs / Fe-Co doped TNTs (**a**); HRTEM images (**b**) low and (**c**) high magnification coupled with SAED.

Sensors surface characterization plays an important role in ion selective electrode (ISE)^47,48^. Figure 3a shows the SEM image of MWCNTs/Fe-Co doped TNTs nanocomposite-based sensor (sensor III).

Unfortunately, no nanotubes were observed on the surface of the graphite sheet. This behavior can be attributed to the magnification and limited resolution of SEM. Consequently, the same paste was checked using HRTEM (Fig. 3b). Well distribution of both nanotubes was observed on the surface and between the graphite layers. The HRTEM in the magnified image (Fig. 3c) confirmed the successful substitution of Na of titanate nanotubes by Fe and Co as indicated in the yellow circles. The selected area electron diffraction (SAED) inserted in HRTEM image (Fig. 3) indicated by spotted rings and the indexing of the few spots with inter planar *d*-spacing values 0.33, 0.2, and 0.12 nm matches with the indices of the planes obtained from XRD analysis which demonstrated the polycrystalline nature of prepared MWCNTs/Fe-Co doped TNTs composite.

**Figure 4:**
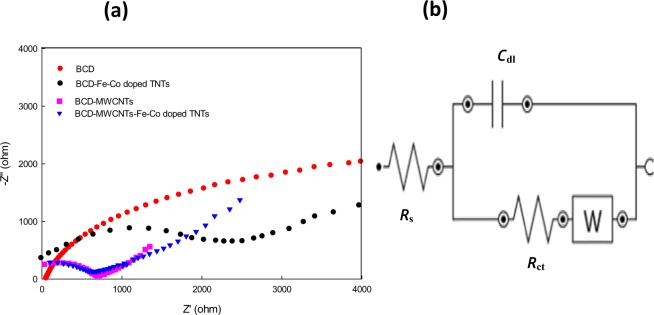
(**a**) Nyquist plots for the investigated sensors in 1.0 × 10^−3^ mol L^−1^ [Fe(CN)_6_]^−3/−4^ containing 0.1 mol L^−1^ KNO_3_ and (**b**) Equivalent circuit.

In order to clarify how the proposed nanomaterials can affect the electrical resistance of CPSs, EIS was carried out. Figure 4a shows EIS of β-CD, β-CD/Fe-Co doped TNTs, β-CD/MWCNTs and β-CD/MWCNTs/Fe-Co doped TNTs measured in solution of 1.0 × 10^−3^ mol L^−1^ [Fe(CN)_6_]^−3/−4^ containing 0.1 mol L^−1^ KNO_3_ and applied at 10 mV amplitude, 1 V vs Ag/AgCl and the frequency extended from 100 kHz to 0.1 Hz. The equivalent circuit reflected the electrical properties of the sensor/solution interface (Fig. 4b). Fitting the electrochemical impedance spectra to the equivalent circuit is responsible on each electrical element value. The diameter of the semicircle decreased from 2.03 kΩ (for β-CD) to 1.36 kΩ (for β-CD/Fe-Co doped TNTs)- Fe and Co were used as dopants for TNTs to enhance the surface area and producing electrons may by through oxidation the SLP as an electron donor^49^ at the sensor surface which enhanced sensor sensitivity to SLP micro determination-to 1.098 kΩ (for β-CD/MWCNTs) to 1.097 kΩ (for β-CD/ MWCNTs/ Fe-Co doped TNTs). This indicated that the *R*_ct_ decreased by adding the MWCNTs/ Fe-Co doped TNTs nanocomposite into the paste. This behavior can be attributed to MWCNTs and Fe-Co doping which can facilitate the electron transfer at the sensor/SLP interface causing improvement in the sensor potential response.

### pH effect

SPECIES program ^50^ was applied to investigate the ionization equilibrium of SLP. Fig. S4a showed that the K_a_ of SLP = 1.3 × 10^− 9^ and at pH 7.4 the drug will be in protonated form.

Sensor (III) was tested to study the effect of pH on the potential values for a 1.0 × 10^−5^, 1.0 × 10^−4^, and 1.0 × 10^−3^ mol L^−1^ SLP solutions. This study revealed that the sensor can be used successfully over a wide pH range 2–8 as shown in Fig. S4b.

### Temperature effect

The performance characteristic of the sensor (III) was examined at different temperatures (17–55) °C. The data revealed that the sensor has a good thermal temperature coefficient (0.0026 V/ ^0^C); demonstrating great immutability of its response during temperature changes. This can be attributed to the presence of MWCNTs and Fe-Co doping TNTs which enhanced sensor thermal stability within the temperature range studied.

### Response time and lifespan

The response time was recorded at different SLP concentrations, over concentration range 1.0 × 10^−7^ to 1.0 × 10^−2^ mol L^−1^. The results indicated that the response time was 5, 7, and 4 s for sensors (I), (II), and (III), respectively as shown in Fig. S5a. The presence of MWCNTs/Fe-Co doped TNTs composite in corporation of sensor (III) plays a vital role in decreasing the resistance, facilitating the electron transfer at the sensor/SLP interface and rapid response time.

The sensor reversibility was checked by a similar procedure in the opposite direction and the results showed that the sensors response was reversible.

Five independent potentiometric sensors have been used to investigate the reproducibility of sensor. The sensor (III) showed good reproducibility with R. S. D less than 1.9%. This may be attributed to presence of MWCNTs/Fe-Co doped TNTs nanocomposite which enhanced the reproducibility and stability of the paste sensor.

The selected sensor (III) is also used to investigate the repeatability. The sensor achieved excellent precision which can be attributed to the low R. S. D. value (0.7%) for three measurements indicating that the sensor had no memory effect (Fig. S5b).

### Interference study

**Figure 5:**
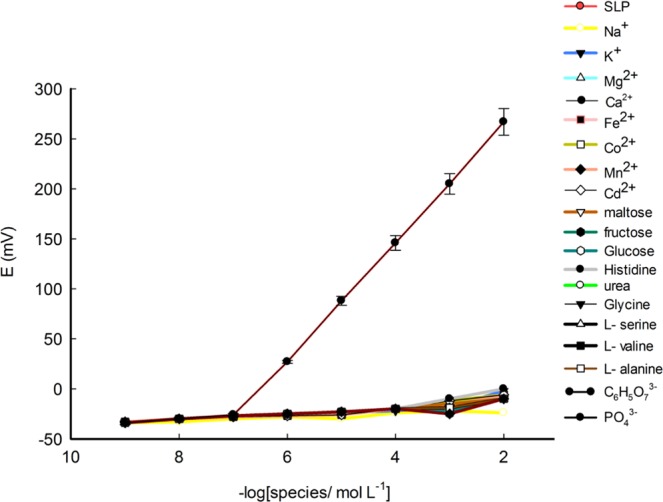
Response to SLP and some interfering species using sensor III.

Selectivity behavior plays an important role which differentiates between the SLP drug against interfering ions^51^. The potentiometric selectivity coefficient values of the sensor (III) were determined by the separate solution method^52^ for some inorganic cations (Na^+^, K^+^, Mg^2+^, Ca^2+^, Cd^2+^, Co^2+^, Mn^2+^, and Fe^2+^) and the matched potential method (MPM)^53^ for organic species, citrate, phosphate, and other pharmaceuticals as shown in Table S1. The results revealed that the sensor is considerably selective to SLP ions in the presence of the interfering species. The selectivity behavior of the sensor was confirmed applying Bakker protocol^54^ as shown in Fig. 5.

### Analytical applications

Sensor (III) was used as an indicator sensor for potentiometric titrations of 2–6 mL of 1.0 × 10^−2^ mol L^−1^ SLP with 1.0 × 10^−2^ mol L^−1^ NaTPB solution as titrant (Fig. S6). Also, the standard addition method was applied successfully for SLP micro determination in pure solutions, pharmaceutical products, spiked urine, and real water samples. The results (Tables S2 and S3) revealed that recoveries ranged from 98.8 to 101.5% with accepted RSD values. F-and t-tests were examined and their values confirmed high precision and accuracy of the proposed sensor.

#### Comparison with reported sensors

CPSs^17^ were used to overcome the inherent limitations of PVC membrane sensor based on ion-pair^55^. Ion-pairs based sensors are usually blocked by limited selectivity and their application are restricted to more challenging biological samples. Consequently, the sensor sensitivity and selectivity were enhanced through SLP/β-CD inclusion complex. The proposed sensor (III) showed a wide LR 1.0 × 10^−7^- 1.0 × 10^−2^ and lower DL 8.7 × 10^−8^ mol L^−1^ compared with other published sensors (Table 2).

**Table 2:** Comparison between the investigated and published sensors.

Sensors	DL (molL^−1^)	LR (molL^−1^)	Slope (mVdecade^−1^)	Response time(s)	Lifetime (weeks)	Ref.
PVC ISE	4.2 × 10^−5^	1.0 × 10^−4^–1.0 × 10^−2^	58.4 ± 0.9	<15	≥ 2	^55^
MWCPE	3.5 × 10^−7^	1.0 × 10^−6^–1.0 × 10^−2^	59.0 ± 0.7	5	22	^17^
β-CDCPE	5.0 × 10^−6^	1.0 × 10^−5^–1.0 × 10^−2^	59.5 ± 0.3	10	17	^17^
Sensor I	7.6 × 10^−7^	1.0 × 10^−6^–1.0 × 10^−2^	57.1 ± 0.4	5	20	[C.S.]
Sensor II	1.58 × 10^−6^	4.0 × 10^−6^–1.0 × 10^−2^	56.0 ± 0.5	7	18	[C.S.]
Sensor III	8.7 × 10^−8^	1.0 × 10^−7^–1.0 × 10^−2^	58.8 ± 0.2	4	25	[C.S.]

C.S.: current study.

## Conclusion

The present work aims to fabricate a novel sensor based on β-CD/ MWCNTs/ Fe-Co doped TNTs (sensor III) for SLP micro determination. The sensor showed high sensitivity, thermal stability, robustness and adequate selectivity. Remarkable enhancement in performance characteristics of the sensor (III) can be ascribed to the excellent properties of nanocomposites. FESEM and HRTEM were used to characterize the structure of the new composite. EIS showed that decreasing the resistance caused improvement of sensor potential reading. Sensor (III) displayed a low DL 8.7 × 10^−8^ mol L^−1^, wide LR (1.0 × 10^−7^ –1.0 × 10^−2^ mol L^−1^), long lifetime (25 weeks) and fast response (4 s). The effect of temperature demonstrated that the novel sensor has good potential stability within the temperature range of 17–55 °C. Moreover; the fabricated sensor has been applied to SLP determination in the real samples with satisfactory results.

## Supplementary information

Supplimentary information.
