# Supplementary material for: Preparation and characterization of novel MWCNTs/Fe-Co doped TNTs nanocomposite for potentiometric determination of sulpiride in real water samples
Source: Sci Rep. 2020 May 25;10:8607. doi: 10.1038/s41598-020-65592-y (PMC7248080; doi:10.1038/s41598-020-65592-y)
Supplement: Supplementary file 1 — Supplimentary information. [file 41598_2020_65592_MOESM1_ESM.docx]

**Preparation and** **characterization** **of** **novel MWCNTs/Fe-Co doped TNTs nanocomposite for potentiometric determination of sulpiride in real water samples**

M. M. Khalil*^,a^, A.A. Farghali^b^, Waleed M. A. El Rouby^b^ and I. H. Abd-Elgawad^a^

^a^ M. M. Khalil

Email: magdy_mmagdy@yahoo.com Institution: Faculty of Science, Beni-Suef University, Beni-Suef, Egypt

Department: Chemistry

^b^A.A. Farghali

Email:ahmedfarghali74@yahoo.com

Institution: Faculty of Postgraduate Studies for Advanced Science, Beni-Suef University, Beni-Suef, Egypt

Department: Materials Science and Nanotechnology

^b^Waleed M. A. El Rouby

Email: [waleedmohamedali@psas.bsu.edu.eg](mailto:waleedmohamedali@psas.bsu.edu.eg)

Institution: Faculty of Postgraduate Studies for Advanced Science, Beni-Suef University, Beni-Suef, Egypt

Department: Materials Science and Nanotechnology

^a^ I. H. A bd-Elgawad

Email: [esslam_hssn@yahoo.com](mailto:esslam_hssn@yahoo.com)

Institution: Faculty of Science, Beni-Suef University, Beni-Suef, Egypt

Department: Chemistry


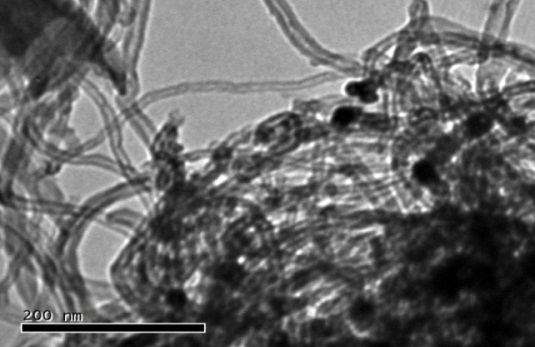


**Fig. S1** HRTEM image of MWCNTs


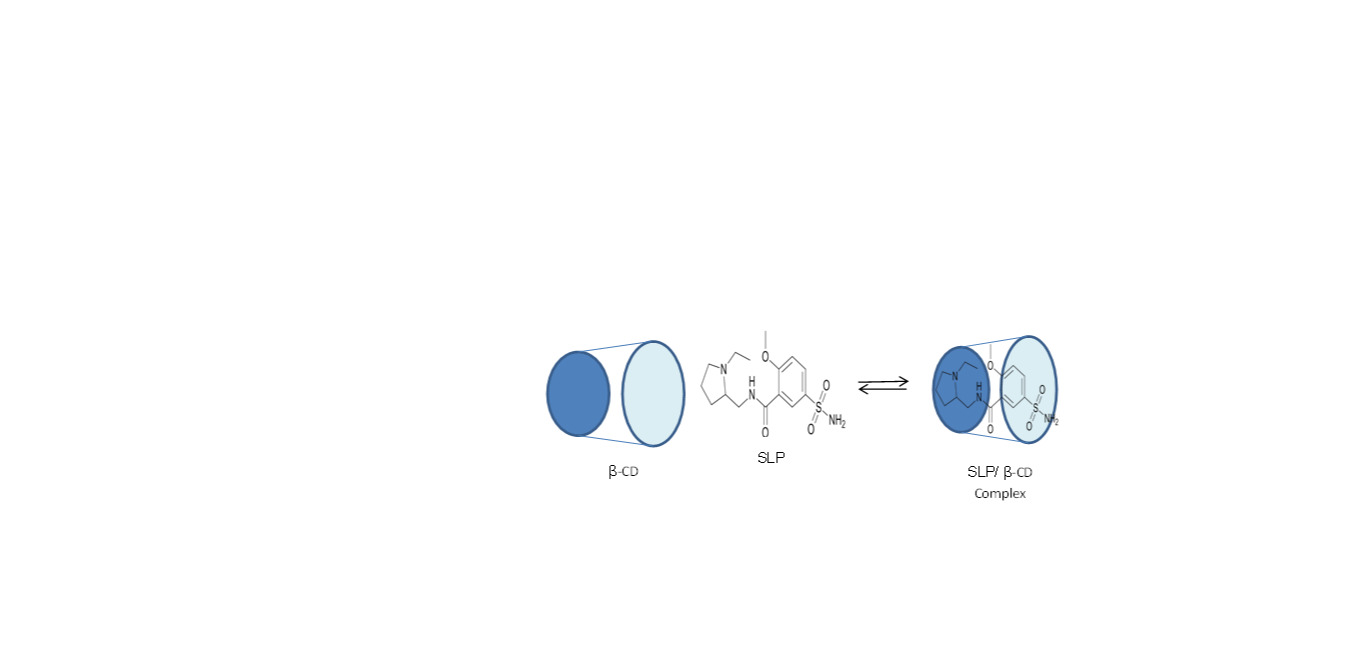


**Fig. S2** Mechanism of SLP/β-CD


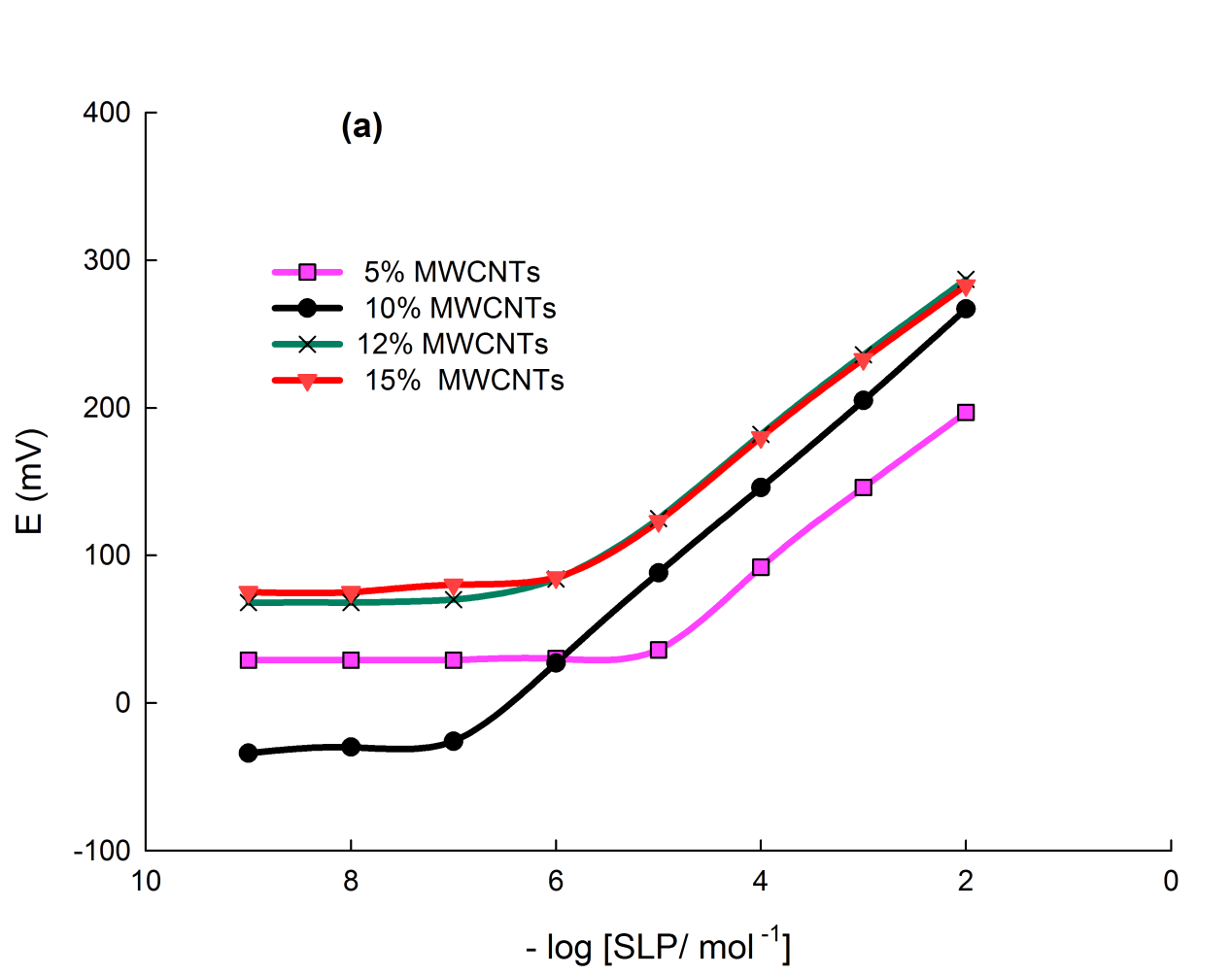

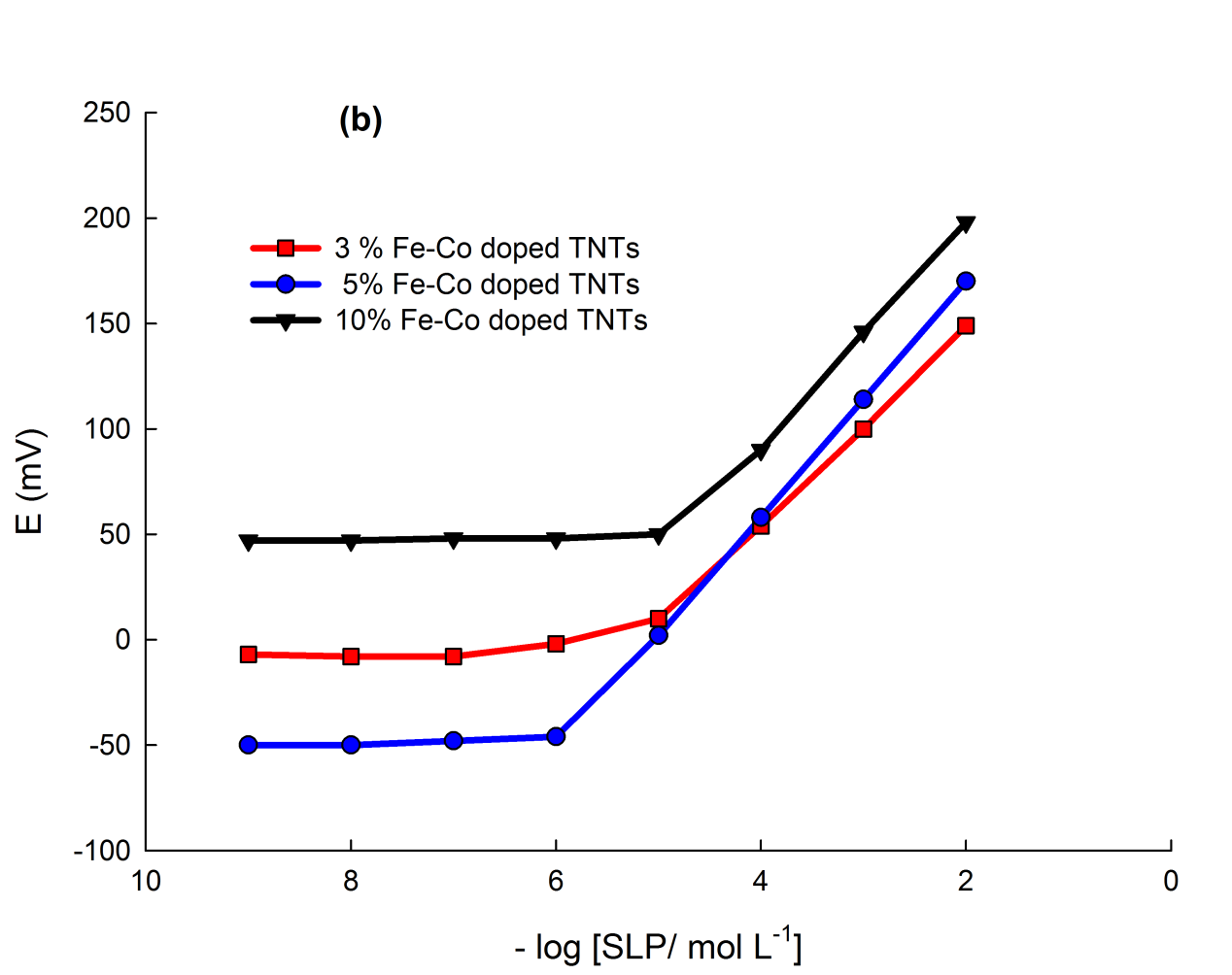


**Fig. S3** Effect of (a) MWCNTs and (b) Fe-Co doped TNTs ratios on SLP sensor performance

**(a)**


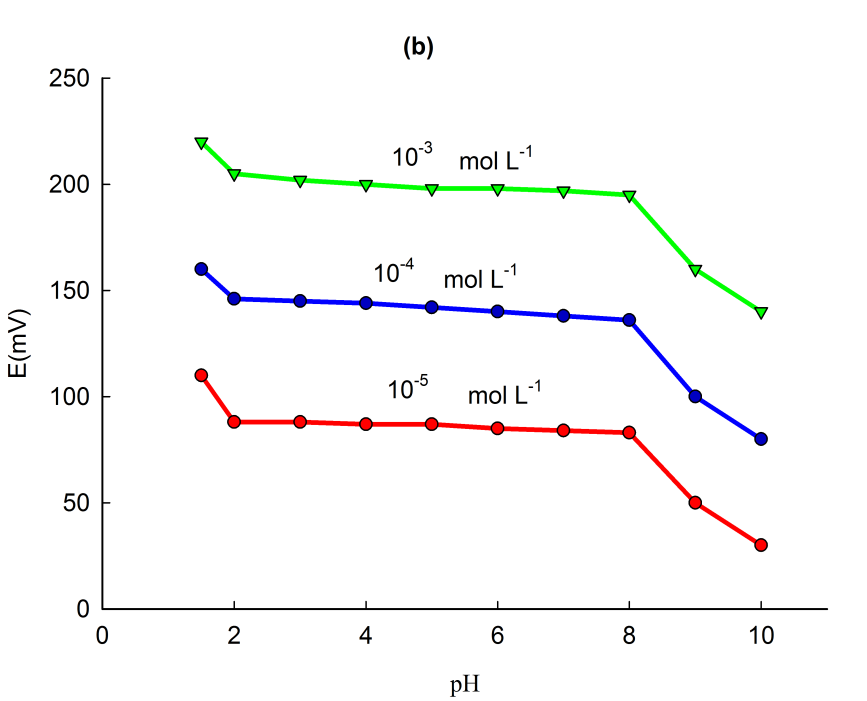


**Fig. S4** (a) Concentration distribution diagram for SLP species and (b) effect of pH at different SLP concentrations on the potential values using sensor III


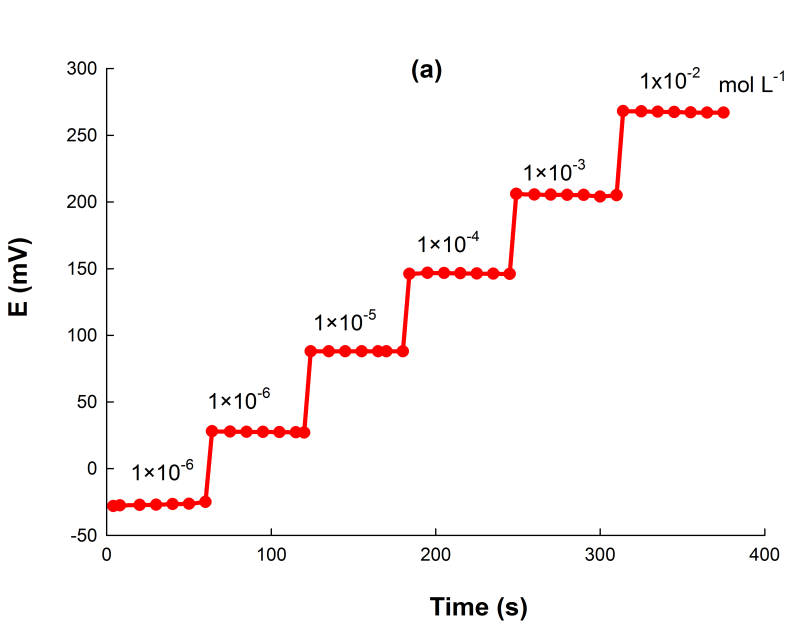

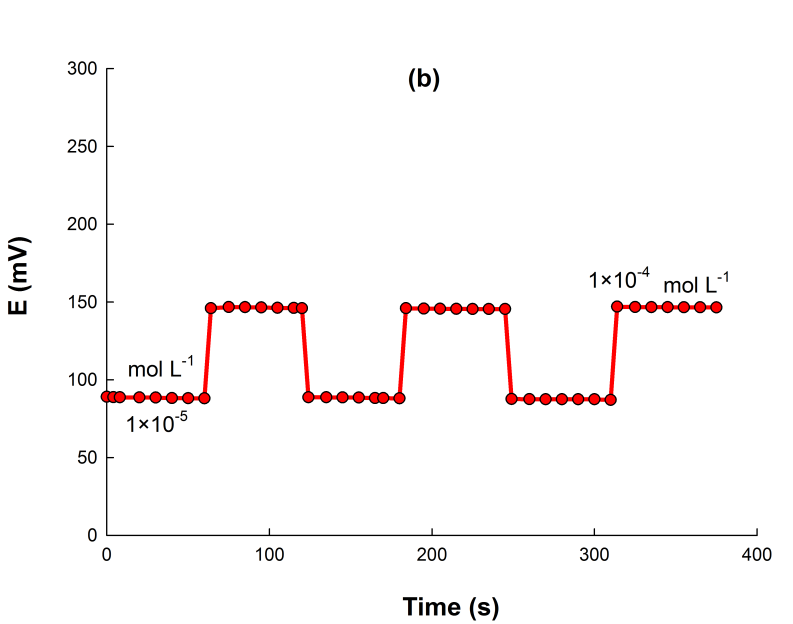


**Fig. S5** (a) Dynamic response time of sensor III and (b) for step change in concentrations of SLP from low to high

**(a)**


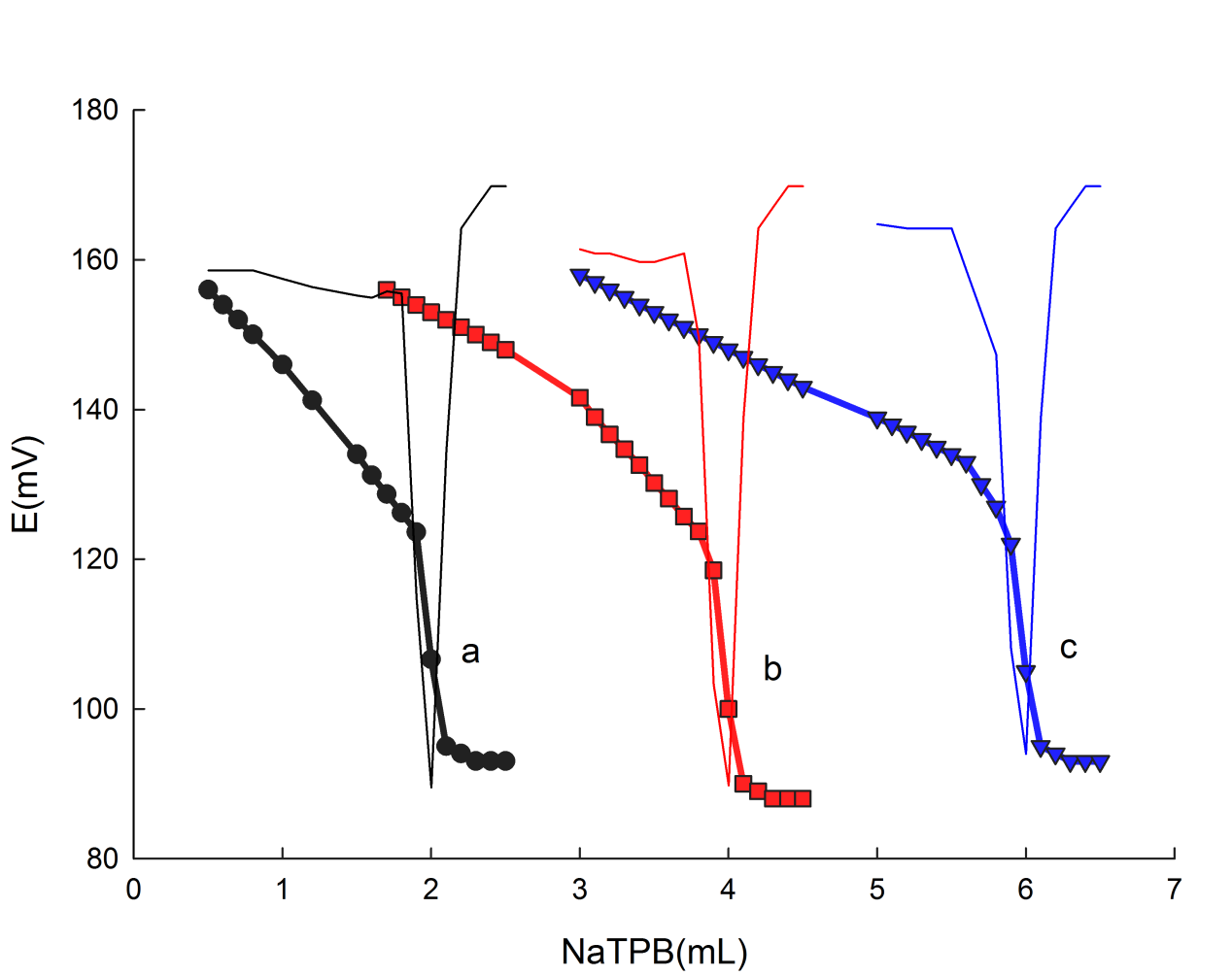


**Fig. S6** Potentiometric titration curves and its first order derivative of (a) 2 (b)4 and (c) 6 mL of 10^-2^ mol L^-1^ SLP using sensor III against 1.0×10^-2^ mol L^-1^ NaTPB as titrant

**Table S1** Selectivity coefficient values for sensor III

|  |  |  |
| --- | --- | --- |
|  | Interfering species | $\log K_{SLP,j}^{pot}$ |
|  |  |  |
|  | Na^+^ | - 3.86 |
|  | K^+^ | - 3.45 |
|  | Mg^2+^ | - 4.20 |
|  | Ca^2+^ | - 3.56 |
|  | Cd^2+^ | -4.22 |
|  | Co^2+^ | -4.21 |
|  | Mn^2+^ | -4.23 |
|  | Fe^2+^ | -4.25 |
|  | Maltose | -3.3 |
|  | Fructose | -3.0 |
|  | Glucose | -3.5 |
|  | Hestidine | -2.4 |
|  | Urea | -3.0 |
|  | Glycine | -3.7 |
|  | L-Serine | -3.2 |
|  | L-Valine | -3.1 |
|  | L-Alanine | -3.2 |
|  | C_6_H_5_O_7_^3-^ | -3.0 |
|  | PO_4_^3-^ | -3.9 |
|  |  |  |

**Table S2** Application of sensor III for the SLP determination in pure and pharmaceutical preparations

| Statistical parameter | Standard addition | | |  | Potentiometric titration | | |
| --- | --- | --- | --- | --- | --- | --- | --- |
|  | Taken (mg) | Recovery % | RSD% |  | Taken (mg) | Recovery % | RSD% |
| Pure solution | 6.83 | 98.8 | 1.7 |  | 6.83 | 99.5 | 1.8 |
|  | 13.66 | 99 | 1.5 |  | 13.66 | 100 | 1 |
|  | 20.48 | 99.5 | 1.3 |  | 20.48 | 101 | 0.49 |
|  |  |  |  |  |  |  |  |
| Mean ± SD |  | 99.1± 1.5 |  |  |  | 100.2±1.1 |  |
| F- ratio |  | 1.36(9.2)^c^ |  |  |  |  |  |
| t-test |  | 2.4(2.78)^e^ |  |  |  |  |  |
|  |  |  |  |  |  |  |  |
| Dogmatil^®^ fort (200 mg per tablet) | 6.83 | 101 | 1.3 |  | 6.83 | 99.8 | 1.28 |
|  | 13.66 | 100 | 1.4 |  | 13.66 | 100.5 | 1.2 |
|  | 20.48 | 100.5 | 1.3 |  | 20.48 | 101.5 | 1.4 |
|  |  |  |  |  |  |  |  |
| Mean ± SD |  | 100.5± 1.3 |  |  |  | 100.6±1.3 |  |
| F- ratio |  | 1.00(9.2)^c^ |  |  |  |  |  |
| t-test |  | 2.5(2.78)^e^ |  |  |  |  |  |

^a^ Tabulated F value at 95% confidence value. ^b^ Tabulated t-value at 95% confidence value and four degrees of freedom

**Table S3** Application of sensor III for the SLP determination in urine and real surface water samples

| Statistical parameter | Taken (mg) | Recovery % | RSD% |
| --- | --- | --- | --- |
|  |  |  |  |
| Urine sample | 6.83 | 99.1 | 0.8 |
|  | 13.66 | 99.8 | 1.2 |
|  | 20.48 | 100 | 1.3 |
|  |  |  |  |
| Mean ± SD |  | 99.63±1.1 |  |
| Surface water | 6.83 | 98.9 | 1.1 |
|  | 13.66 | 99 | 1.4 |
|  | 20.48 | 99.5 | 1.5 |
|  |  |  |  |
| Mean ± SD |  | 99.13±1.3 |  |
